# Chitosan/PVA/Doxycycline Film and Nanofiber Accelerate Diabetic Wound Healing in a Rat Model

**DOI:** 10.22037/ijpr.2020.112620.13859

**Published:** 2020

**Authors:** Keshvad Hedayatyanfard, Shadab Bagheri Khoulenjani, Mohammad Amin Abdollahifar, Davar Amani, Behnam Habibi, Fatemeh Zare, Ali Asadirad, Ramin Pouriran, Seyed Ali Ziai

**Affiliations:** a *Department of Physiology and Pharmacology, School of Medicine, Alborz University of Medical Sciences, Karaj, Iran. *; b *Cardiovascular Research Center, Alborz University of Medical Sciences, Karaj, Iran. *; c *Polymer and Color Engineering Department Amirkabir University of Technology (Tehran Polytechnic) Tehran, Iran.*; d *Department of Biology and Anatomical Sciences, School of Medicine, Shahid Beheshti University of Medical Sciences, Tehran.*; e *Department of Immunology, School of Medicine, Shahid BeheshtiUniversity of Medical Sciences, Tehran, Iran. *; f *Department of Pharmacology, School of Medicine, Shahid Beheshti University of Medical Sciences, Tehran, Iran. *; g *Department of Immunology, Faculty of Medicine, Ahvaz Jundishapur University of Medical Sciences, Ahvaz, Iran.*

**Keywords:** Chitosan, Doxycycline, Film, Nanofiber, Diabetic wound, Polyvinyl alcohol

## Abstract

In this study, we evaluated the effects of nanofiber and film polymers with doxycycline for treating a wound in a diabetic rat model. 108 male rats were divided into six groups, the control group, the diabetic control, and the groups were diabetic rats receiving different wound dressing. At the 3rd, 7th, and 14th days, macroscopic/histologic imaging and tissue sampling were performed. Tissues were analyzed for IL-1β, TNF-α, IL-10, TIMP-1, and MMP-2 by using ELISA. Dressings of chitosan, polyvinyl alcohol, and doxycycline increased the rate of wound closure, the volume of collagen, dermal, and epidermis. In addition, it increased the number of fibroblasts and basal cell epidermis cells, vascular length, and decreased the number of neutrophil cells. Inflammatory cytokines and MMP-2 were decreased, and anti-inflammatory IL-10 and TIMP-1 were increased. It was ultimately attained that the combination of chitosan/ polyvinyl alcohol /doxycycline could be a useful dressing for the healing of diabetic wounds.

## Introduction

Skin is the largest organ in the human body and consists of three layers of epidermis, dermis, and hypodermis (subcutaneous tissue) (1). Wound healing includes several stages, such as homeostasis, inflammation, proliferation, and remodeling (2). Diabetes is one of the most costly and common diseases worldwide as of now. Approximately, 15% of diabetic patients develop diabetic foot ulcers (3). Chronic diabetic ulcers are difficult to treat and impose a cost burden on the patient and the health care system in the community. Generally, in comparison with the acute ulcer, a chronic ulcer contains a large concentration of IL-1, IL-6, and TNF-α. According to the data, the level of pro-inflammatory cytokines (TNF-α, IL-1β) in chronic ulcers were about 100 times more than acute ulcers; on the other hand, the mitogenic activity, division rate, and cell differentiation were significantly lower in the reports (4). One of the effective treatments for chronic ulcers is the disruption of the pro-inflammatory cycle (5). The matrix metalloproteinase (MMP) is ​​a zinc-dependent protease and can degrade the entire extracellular matrix compounds (6). Normally, the tissue fibroblast inhibitors called (tissue inhibitor of metalloproteinase) TIMP-1, 2, 3, and 4 exist in the human body tissues (7). The two indispensable MMPs are Gelatinase A (MMP-2) and Gelatinase B (MMP-9), which could degrade the entire active forms of the extracellular matrix (ECM) collagen (8). Pro-inflammatory cytokines can play a major role in the regulation and expression of MMPs, especially MMP-2,9. One of the cytokines known to have an anti-inflammatory role is interleukin-10 (IL-10) (9). Distinctly, IL-10 has been shown to stimulate TIMP-1 secretion and inhibit the secretion of MMP-2 and MMP-9 by prostate tumor cells (10). It also exerts inhibitory effects on pro-inflammatory cytokines (9). Chitosan is a natural biocompatible, biodegradable, non-antigenic polymer with anti-microbial effects and is obtained from chitin (11, 12). Studies have shown that chitosan has coagulation, antioxidant, and wound-healing properties (13-17). Polyvinyl alcohol is a synthetic polymer used in the pharmaceutical and cosmetic industries (18). Doxycycline is a synthetic broad-spectrum antibiotic in the tetracycline family (19, 20). In addition to acting as antibiotics, tetracyclines can also affect inflammation, regulation of the immune system, cell proliferation, and angiogenesis (21-24). Various studies have indicated that doxycycline has been capable in inhibit pro-inflammatory cytokines, and MMPs (25). Results of a study in 2009 showed that doxycycline inhibited IL-1β mRNA and decreased its bioactivity (26). Doxycycline also led to a significant inhibition in the production of TNF-α, IL-6, IL-8, and down-regulation of the NF-κB gene (27). In one study, it was revealed that this drug significantly inhibited IL-1β and TNF-α cytokines (28). Electrospinning is a method, in which electrostatic forces are used to produce nanofibers from polymeric solutions (29). These types of wound dressing improve and enhance cell-cell and matrix-cell responses along with the gene expression and normal phenotypic shape of cells (30). Evaluation and determination of the effect of doxycycline and chitosan in chronic wound healing is the novelty of the present study. In our pervious study, we evaluated the physiochemical (scanning electron microscope (SEM), Fourier-transform infrared spectroscopy (FTIR spectroscopy), dissolution test, permeation test, swelling test, agar diffusion antibiogram) of different formulation of films and nanofibers based on chitosan/polyvinyl alcohol/Doxycycline 1% with and whitout Genipin (crosslinker). We found that the chitosan/polyvinyl alcohol/Doxycycline 1% film and nanofiber had better release *in-vitro* studies for films and nanofiber wound dressing. So, this formulation was selected for this study (31). The present study was aimed to compare the effect of two film and nanofiber formulations containing chitosan, polyvinyl alcohol, and doxycycline on the wound healing rate, concentration of Interleukin-1β (IL-1β), tumor necrosis factor (TNF-α), Interleukin-10 (IL-10), matrix metalloproteinase (MMP-2), tissue inhibitor of metalloproteinase (TIMP-1), and histology and tissue stereology in the wound site in a model of diabetic rat. 

## Experimental

*Diabetic model*

A total of 108 adult Wistar male rats, weighing 200-250 g were randomly selected and divided into 6 groups (control, diabetic, nanofiber Chitosan/Polyvinyl alcohol, nanofiber Chitosan/Polyvinyl alcohol/Doxycycline, film Chitosan/Polyvinyl alcohol, and film Chitosan/Polyvinyl alcohol/Doxycycline), sacrificed and studied in 3 separated time (3rd, 7th, and 14th day). Each group-time contained 6 rats. The Shahid Beheshti University of Medical Sciences Ethics Committee approved the animal experiment (SBMU.REC 1393.334). All the rats were kept in an especial cage and had free access to water and food. They were placed at 25 °C and 12:12 h light-dark cycle. Streptozotocin (60 mg/kg) (Sigma) was used to induce diabetes. Primarily, pure streptozotocin powder was mixed with 500 μL of fresh sodium citrate solvent at 4 °C in dark conditions and then placed inside the microtube containing aluminum cover and intraperitoneally injected into 5 groups of 18 rats. The sixth group was considered as a non-diabetic control group. The cages were controlled in terms of access to water and food. The blood glucose sampling was conducted in the tail vein with a glucometer after one week of drug injection. Diabetic levels were considered to be over 300 mg/dL of blood glucose. Fourteen days after induction of diabetes, all the rats were anesthetized by using ketamine-xylazine. An electric shaver was used to shave the hair on the back region of the rat skin and the surgical instruments disinfected by 70% alcohol. In each rat, a 15 mm wound was made in the back of the animal by removing the skin. It should be noted that each group of 18 rats was divided into three subgroups (n = 6 per subgroup) to carry out macroscopic, microscopic, and immunological evaluations on days 3, 7, and 14 after diabetization.

*Digital imaging*

In order to determine the wound healing rate, digital imaging was performed on the wound area on the first day and on the day when sampling was carried; eventually, the rats were sacrificed. A digital software (Medclac software) Digimizer was used to examine and analyze the percentage of reduction in the wound surface area compared to day zero.

*Dressing preparation*

Two different films (f) formulations: f-Chitosan (C)/polyvinyl alcohol (P) and f-C/P/Doxycycline (D) 1% (Sigma Aldrich Co; USA) were fabricated by the casting method. Chitosan (low molecular weight, 75–85% degree of deacetylation) powder 3% (w/v) was dispersed in acetic acid 1% for 3 h on stirrer with 250 rpm at 25 °C. Polyvinyl alcohol (M_w_ 89000-98000, 99% hydrolyzed) powder 5% (w/v) was dispersed in distilled water 120 °C with intense stirring for 3 h. After degassing, C/P solutions 20/80 (v/v) were mixed for 18 h at 25 °C. Doxycycline 1% (w/w) was dissolved in distilled water and added to the chitosan / polyvinyl alcohol solution.

For nanofiber preparations: Chitosan (low molecular weight, 75–85% degree of deacetylation) powder 3% (w/v) was stirred with 250 rpm in acetic acid 70% (v/v) for 3 h at 25 °C. Polyvinyl alcohol (M_w_ 89000-98000, 99% hydrolyzed) powder 10% (w/v) was dispersed in distilled water at 120 °C with intense stirring for 3 h. After degassing the solutions chitosan and polyvinyl alcohol with the ratio of 20/80 (v/v) were mixed overnight for 18 h at 25 °C., Doxycycline 1% (w/w) weredissolved in distilled water and added to chitosan/polyvinyl alcohol solutions. For electrospinning, the polymer solutions were inserted into a 5 mL glass syringe with a stainless steel 19-gauge needle, the distance between the tip and aluminum collector was 15 cm; high voltage power 20 kV was applied and the feed rate for injecting the solution was 0.3 mL/h (31).

*Application of film and nanofiber dressings*

The dressings were carefully placed on glue as the second dressing while maintaining the cleaning conditions of the area. We used the non-woven dressing (Moris Co) as a secondary dressing. Before applying the dressing on the wound, all wound dressing and normal saline were sterilized with UV for 30 min. The above dressings were removed from the rat’s wound site every 24 h and replaced with new dressings under the same conditions.

*Sampling*

In order to sample the wound site and examine the effect and compare the dressings, the rats were sacrificed by using CO_2_ gas in three groups and in the following days (Days 3, 7, and 14). Two specimens were taken from the wound site and the healing of the rats by using surgical instruments: a specimen for histological examination and ELISA was tested. The specimens were immediately taken to the nitrogen tank, then got frozen and transferred to the refrigerator-80 °C.

*Stereology*

**Figure 1:**
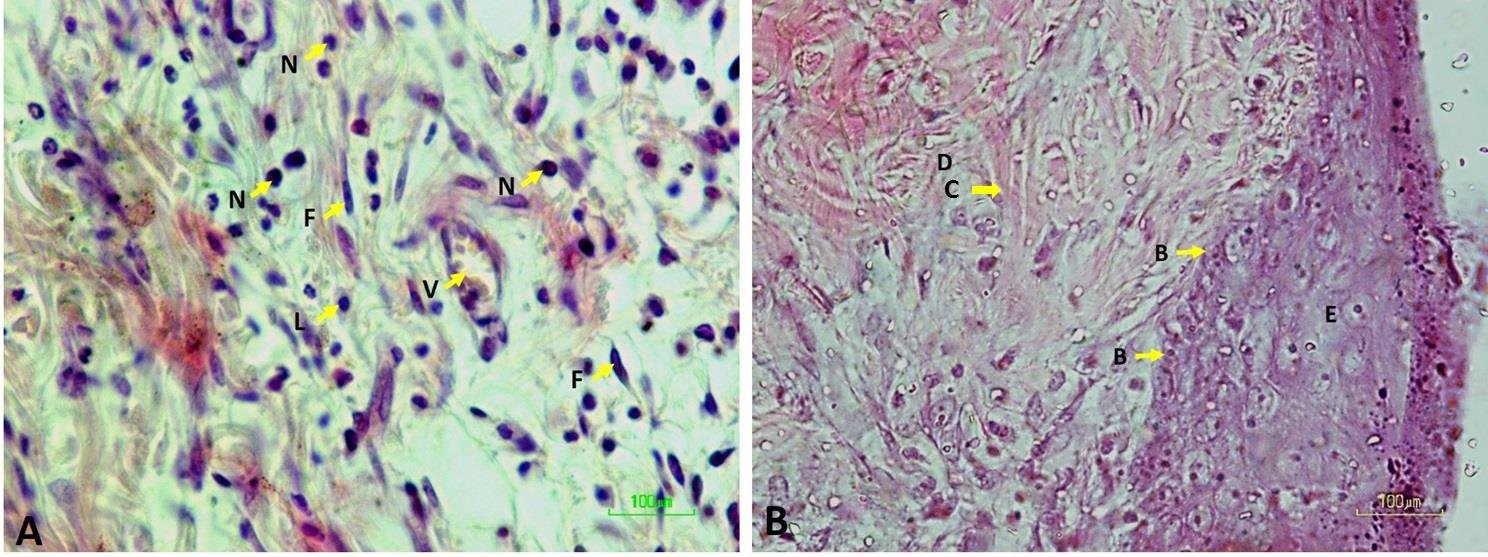
H&E staining of a wound area (E: epiderm, D: derm, B: the basal layer of the epidermis, C: collagen, F: fibroblast, N: neutrophils, and V: vessels) (40X)

The skin samples were immersed in 10% formalin (Merck Germany) for 7 days and then embedded in paraffin block (Merck Germany). Subsequently, serial sections (10 μm thickness) were made, by using a microtome (Leica RM2255 USA). For each rat 8–10 sections were selected in a systematic random manner. All tissue sections were stained with Hematoxylin and Eosin (H&E) and Masson’s Trichrome (Figure 1)

*Estimation of the volume of the new epidermis, dermis, fibrous tissue*

To determine the total volume of the new fibrous tissue, epidermis, and Cavalieri’s principle, the following equation was used (32): 

〖V〗_total = ∑ 〖P×a/p〗×t 

 Equation. 1

t is the distance between the sampled sections. The ΣP is estimated, by using the point-counting method, and the a/p is the area associated with each point projected on the skin tissue (32).

*Estimation of the number of fibroblast, neutrophil and basal cell*

To determine the number of fibroblast and neutrophil, basal cells were performed through the optical dissector method (32). The positions of the microscopic fields were selected by an equal interval of moving the stage and systematic uniform random sampling. Microcator was used for the measurement of the Z-axis movement of the microscope stage. An unbiased counting frame with inclusion and exclusion borders was superimposed on the images of the sections viewed on the monitor. A nucleus was counted if it was placed completely or partially within the counting frame and could not reach the exclusion line. Numerical density (Nv) was calculated by the following formula:

N_v = (∑ Q)/(∑ 〖P×h×a/f〗)×t/BA 

 Equation. 2

In this formula “ΣQ-” is the number of the nuclei 

“h” is the height of the dissector 

“a/f” is the frame area

“ΣP” is the total number of the unbiased counting frame in all fields

 “t” is the section thickness measured in every field, by using the microcator

 BA is the block advance of the microtome, which was set at 10 μm

*Estimation of the length of the vessels*

The length of the vessels was measured, by using the following formula (33).

L_v = (2∑ Q)/(∑ 〖P×a/f〗) 

Equation. 3 

In this formula, “ΣQ” denoted the total number of the vessel profiles counted per skin

“ΣP” and “a/f” were the total number of the counted frame and the area of the counting, respectively.

*ELISA tests*

Primarily, tissue specimens were removed from the freezer -80 °C, homogenized and dimensions with certain weights were poured in the microtube containing the lysis buffer with protease inhibitor. The microtubes were incubated at the refrigerator temperature (4 ˚C) for 1 h and then centrifuged at 14,000 rpm to remove the supernatant. The concentrations of the above compounds were calculated by the ELISA method (MMP-2 My BioSource, San Diego, California, USA) and IL-1β, IL-10, TIMP-1, TNF-α (Ray Biotech Norcross, GA, USA) according to the manufacturer’s instructions. The ELISA test for each sample was performed in duplicate.

*Statistical analysis*

The entire data for the dissection were calculated by using Prism Graph Pad software and the significant differences were calculated at *P *< 0.05. It should be noted that one-way ANOVA was used to compare the differences between the groups.

## Results

*Wound size reduction*

**Figure 2:**
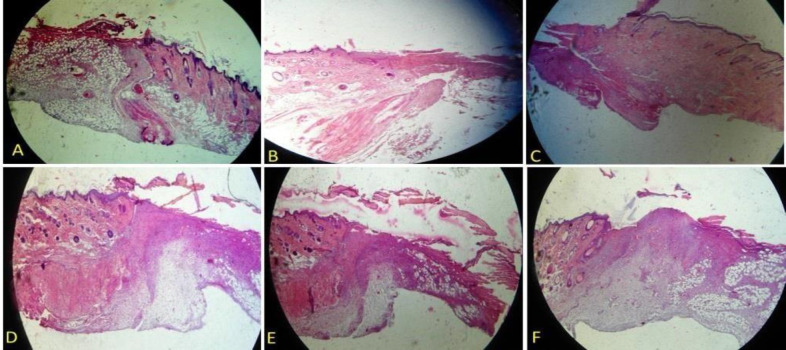
H&E staining of a wound area on days 3. A: Control, B: Control of diabetes, C: nanofiber-Chitosan/Polyvinyl alcohol, D: nanofiber-Chitosan/Polyvinyl alcohol/Doxycycline, and E: film-Chitosan/Polyvinyl alcohol, and F: film-Chitosan/Polyvinyl alcohol/Doxycycline (4X).

**Figure 3:**
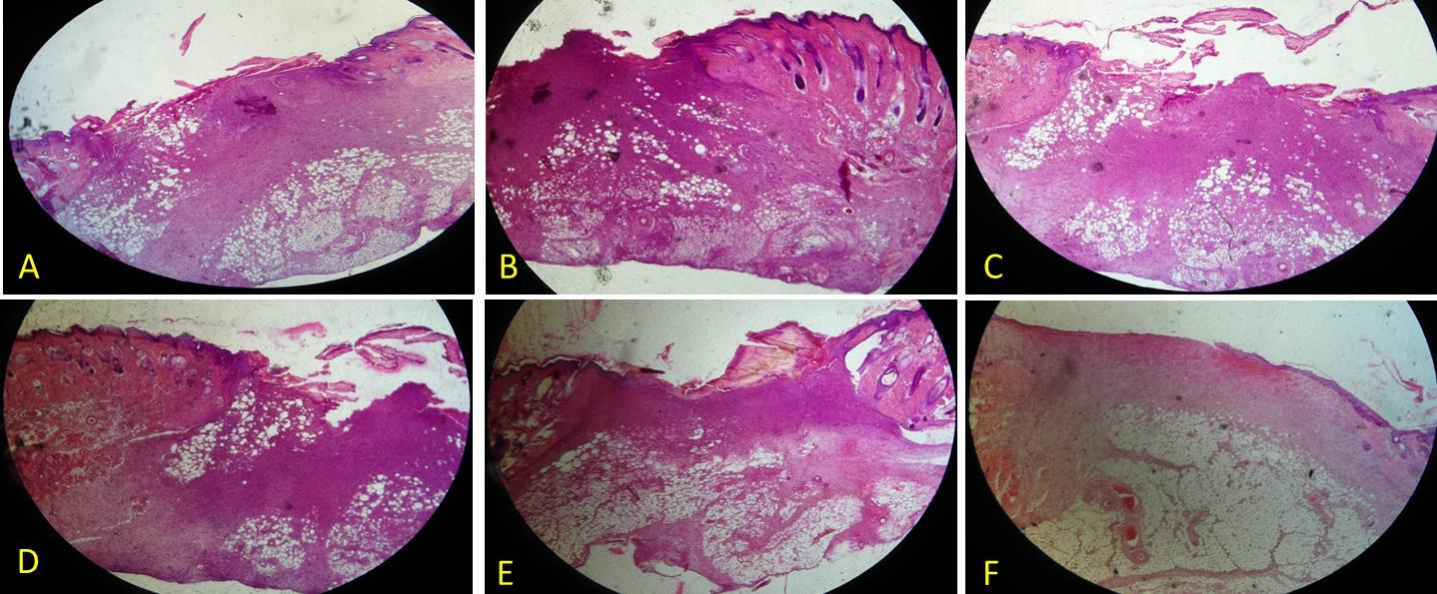
H&E staining of a wound area on days 7. A: Control, B: Control of diabetes, C: nanofiber-Chitosan/Polyvinyl alcohol, D: nanofiber-Chitosan/Polyvinyl alcohol/Doxycycline, and E: film-Chitosan/Polyvinyl alcohol, and F: film-Chitosan/Polyvinyl alcohol/Doxycycline (4X)

**Figure 4:**
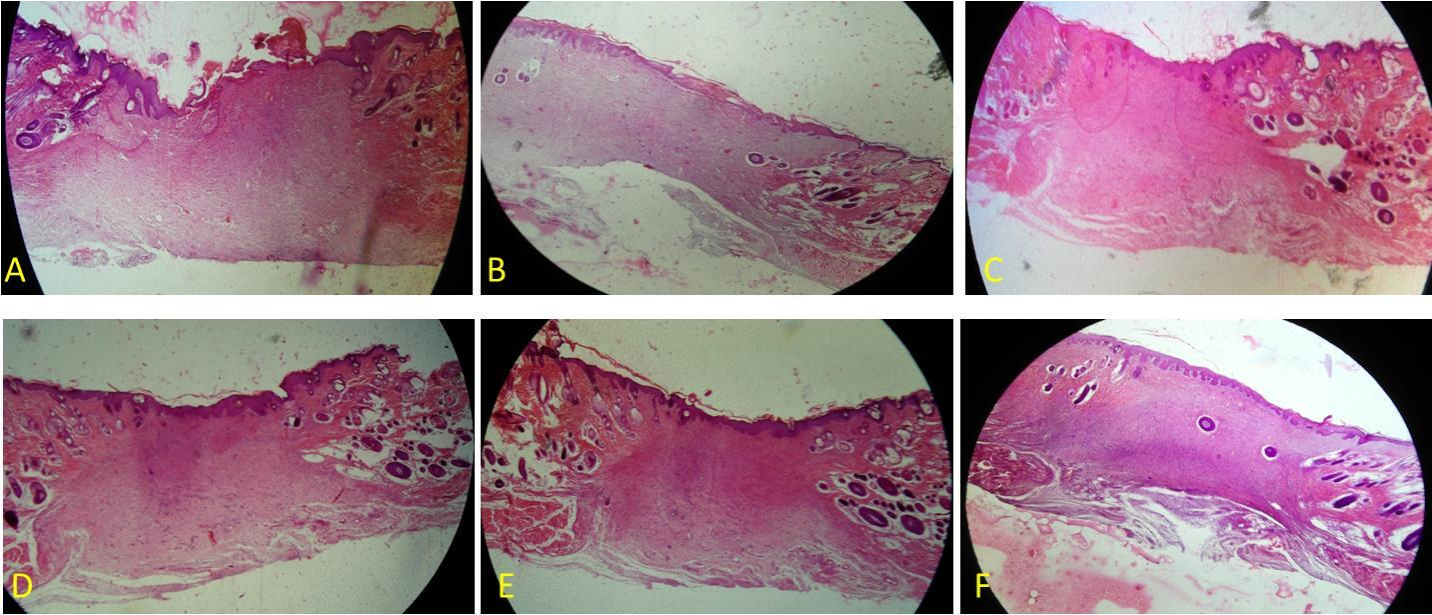
H&E staining of a wound area on days 14. A: Control, B: Control of diabetes, C: nanofiber-Chitosan/Polyvinyl alcohol, D: nanofiber-Chitosan/Polyvinyl alcohol/Doxycycline, and E: film-Chitosan/Polyvinyl alcohol, and F: film-Chitosan/Polyvinyl alcohol/Doxycycline (4X)

**Figure 5:**
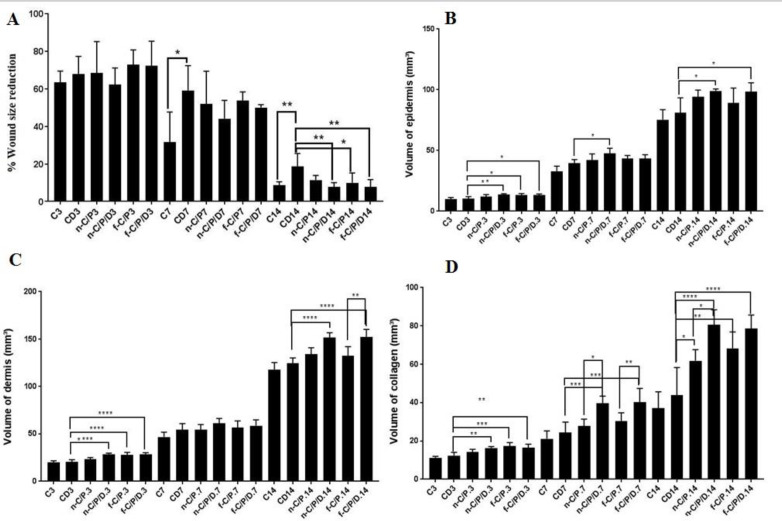
The bar chart Analysis. (A) Percentage reduction of the wound surface, (B) Volume of the epidermis, (C) Volume of dermis and (D) Volume of collagen from the studied groups on days 3, 7 and 14. C: Control, CD: Control of diabetes, n-C/P: nanofiber-Chitosan/Polyvinyl alcohol, n-C/P/D: nanofiber-Chitosan/Polyvinyl alcohol/Doxycycline, f-C/P: film-Chitosan/Polyvinyl alcohol, and f-C/P/D: film-Chitosan/Polyvinyl alcohol/Doxycycline. Significance only showed between dressing and CD groups (*: *P* < 0.05), (**: *P* < 0.01), (***: *P *< 0.001), and (****: *P *< 0.0001)

The findings showed no major histological variations in the six groups (Figures 1, 2, 3 and 4) and showed a decrease in the wound surface area on the third day (Figure 5A). On the seventh day, the wound healing rate was significantly lower in the control diabetic group (CD7) than the control group (C7) (*P* = 0.0105). Additionally, the wound surface area was lower in all dressing groups than the CD group and the groups receiving doxycycline dressing than the non-doxycycline one; nonetheless, this difference was not statistically significant. At the end of the day 14, the groups receiving doxycycline film and nanofiber formulation such as n-C/P/D14 (*P* = 0.0041) and f-C/P/D14 (*P* = 0.0027) led to a significant decrease in the wound surface area. The f-C/P 14 film group also showed a significant reduction in the wound surface area compared to the CD group (*P* < 0.05). In addition, doxycycline dressing groups led to a higher reduction in the wound surface area compared to the non-doxycycline dressing groups; nevertheless, this difference was not significant. In addition, the control group (C14) led to a significant wound healing rate as compared to the CD group (CD14) on day 14 (*P* = 0.0087). 

*Volume of epidermal*

The obtained data (Figure 5B) indicated that the n-C/P/D3, f-C/P3, and f-C/P/D3 dressing groups led to a significant increase in the epidermis volume than the control diabetic group (CD3) (*P* < 0.05). On the 7th day of histological evaluation, the n-C/P/D7 group had the most desirable performance in increasing the epidermal volume (47.71 ± 4.092) compared to CD7 (*P* < 0.0001). On the 14th day of histological evaluation, doxycycline nanofiber and film dressing formulations significantly increased the epidermal volume in comparison to the control group (C14) (*P* < 0.01) and the diabetic control group (CD14) (*P* < 0.05). Indeed, the performance of the doxycycline nanofiber dressing group was significantly more desirable than doxycycline film dressing, but it was not sufficient enough.

*Volume of dermis*

The results indicated that the n-C/P/D3, f-C/P/D3 and f-C/P/D3 groups led to a more significant increase in the dermal volume as compared to the CD3 group on the 3rd day (Figure 5C) (*P *< 0.0001). Moreover, n-C/P/D14 and f-C/P/D14 groups significantly increased dermal volume in comparison with the CD14 group on the 14th day (*P *< 0.0001). Also, these groups significantly increased the dermal volume compared to the non-doxycycline film (*P* < 0.001) and non-doxycycline nanofiber dressing groups (*P* = 0.01).

*Volume of collagen*

The results indicated that the n-C/P/D3 (*P* < 0.01), f-C/P3 (*P* < 0.001), and f-C/P/D3 (*P* < 0.01) formulations increased in the collagen volume compared to the CD group (Figure 5D). On the 7th day, the n-C /P/D7, and f-C/P/D7 recipient groups significantly increased the collagen volume compared to the CD7 group (*P *< 0.001). These groups also showed significantly greater collagen volume than the non-doxycycline film (*P *< 0.01) and non-doxycycline nanofiber dressing groups (*P* < 0.05). On the 14th day, all doxycycline film (*P *< 0.0001) and nanofiber dressing (*P *< 0.0001) groups as well as the non-doxycycline film (*P *< 0.01) and nanofiber (*P *< 0.05) increased the collagen volume compared to CD14 group. The obtaining data also revealed that doxycycline film dressing and doxycycline nanofiber dressing had the best performance in increasing the collagen volume on the 7th and 14th day of the study, respectively.

*Number of neutrophils*

**Figure 6.**
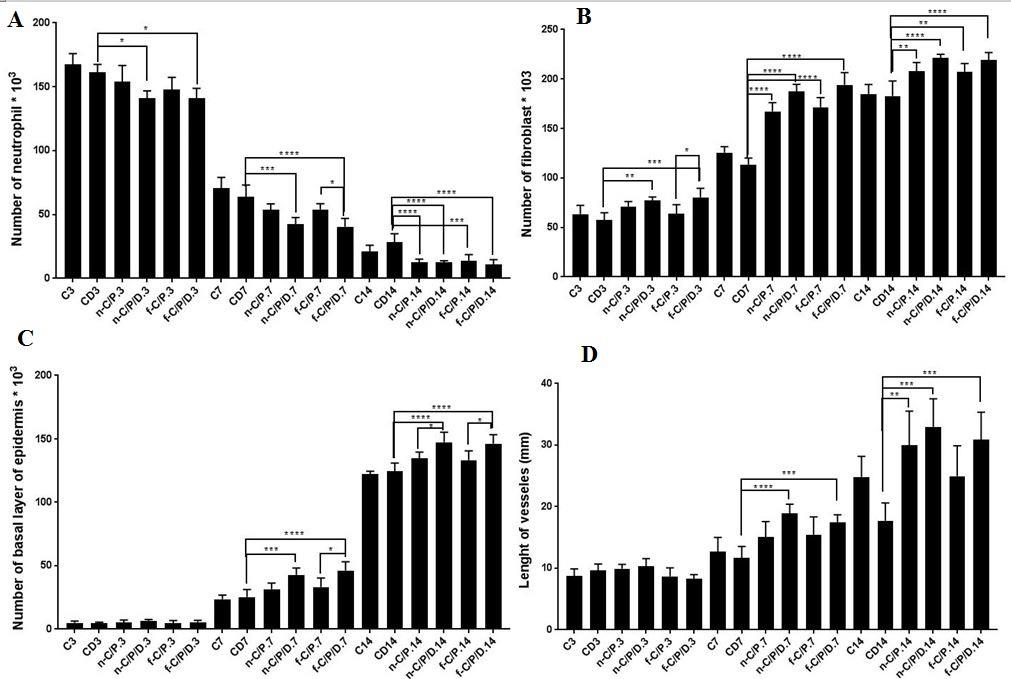
The bar chart Analysis. (A) Number of neutrophils,(B) Number of fibroblasts, (C) Number of the basal layer of the epidermis, and (D) Length of vessels from the studied groups on days 3, 7 and 14. C: Control, CD: Control of diabetes, n-C/P: nanofiber-Chitosan/Polyvinyl alcohol, n-C/P/D: nanofiber-Chitosan/Polyvinyl alcohol/Doxycycline, f-C/P: film-Chitosan/Polyvinyl alcohol, and f-C/P/D: film-Chitosan/Polyvinyl alcohol/Doxycycline. Significance only showed between dressing and CD groups (*: *P* < 0.05), (**: *P* < 0.01), (***: *P *< 0.001), and (****: *P *< 0.0001)

The results showed that the doxycycline film (*P* = 0.0124) and nanofiber dressing (*P* = 0.0125) groups significantly decreased the neutrophil count in the wound site on the 3rd day compared to CD3 (Figure. 6A). Furthermore, the neutrophil count in the CD3 group was lower than the C3 group (*P* > 0.05). On the 7th day, in comparison to the CD7 group, doxycycline film (*P *< 0.0001) and nanofiber dressings (*P* = 0.0002) could significantly reduce the neutrophil count. On the 7th day, the neutrophil count in the CD7 group was lower than the C7 group (*P *> 0.05). On the 14th day, all dressing groups significantly reduced the neutrophil count compared to the CD14 group. Indeed, the doxycycline film formulation exhibited a better performance in reducing the neutrophil count as compared to the nanofiber formulation on the 7th and 14th days. Moreover, the neutrophil count in the CD14 group was higher than the C14 group on the 14th day, (*P* > 0.05). The remarkably greater neutrophil count was present in the CD14 group than the C14 group, while the neutrophil count was higher in the control compared to the CD groups on the 3rd and 7th days (*P *> 0.01).

*Number of fibroblasts*

As the (Figure.6B) shows, doxycycline film (*p* < 0.001), and nanofiber (*p *< 0.01) dressing significantly increased the fibroblast count as compared to the CD3 group; On the 7th day, all dressing groups significantly increased the fibroblast count compared to the CD7 groups. Similarly, on the 14th day, all dressing groups significantly increased the fibroblast count compared to the CD14 group. 

*Number of the basal layer of the epidermis*

Results showed no significant difference between groups on the 3rdday. On the 7th day, all doxycycline film (*P *< 0.0001) and nanofiber dressings (*P *< 0.001) significantly increased the number of basal epidermal cells compared to the CD7 group (Figure 6C). On the same day, the best result was related to the doxycycline dressing groups. On the 14th day, all doxycycline film and nanofiber dressings led to a significant increase in the number of epidermal basal cell (*P *< 0.0001) compared to the CD14 group. In fact, these groups increased significantly the epidermal basal cells compared to the non-doxycycline film and nanofiber dressing groups (*P *< 0.05). On the same day, nanofiber dressings had a better outcome than the film dressing (non-significant).

*Length of vessels*

(Figure 6D) shows no significant difference between the studied groups on the third day. On the seventh day, only doxycycline film and nanofiber dressings significantly increased the length of the vessels compared to the CD7 group. On the 14th day, doxycycline film (*P *< 0.001), nanofiber dressing groups (*P *< 0.001), and non-doxycycline nanofiber dressing group (*P *< 0.01) significantly increased the length of the vessels compared to the CD14 group. Overall, nanofiber dressings have a better result in improving the vessel length compared to film dressings in this experiment.

*IL-1β*

**Figure 7:**
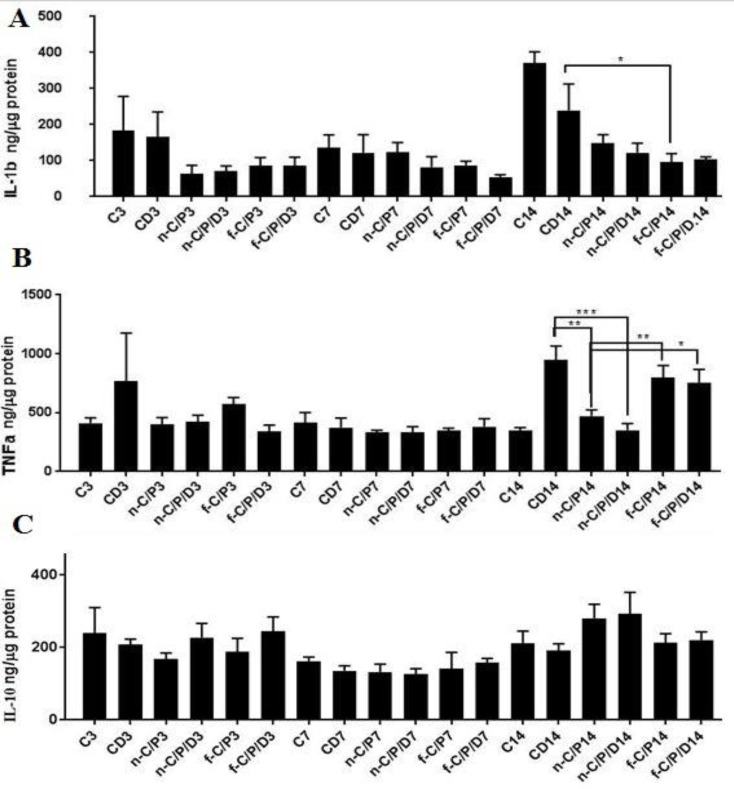
The bar chart Analysis (ELISA). A: IL-1 β, B: TNF-α, C: IL-10 from the studied groups on days 3, 7 and 14. C: Control, CD: Control of diabetes, n-C/P: nanofiber-Chitosan/Polyvinyl alcohol, n-C/P/D: nanofiber-Chitosan/Polyvinyl alcohol/Doxycycline, f-C/P: film-Chitosan/Polyvinyl alcohol, and f-C/P/D: film-Chitosan/Polyvinyl alcohol/Doxycycline. Significance only showed between dressing and CD groups (*: *P* < 0.05), (**: *P* < 0.01), and (***: *P *< 0.001)

According to (Figure 7A), in the 3rd day, there was no significant difference between the groups despite the decrease in the interleukin-1 beta (IL-1β) concentration in all groups receiving film and nanofiber dressings, compared to the C3 and CD3 groups. On the 7th day, doxycycline film and nanofiber dressing groups reduced IL-1β concentration compared to non-doxycycline groups, C7, and CD7 groups; nonetheless, this difference was not significant. On the 14th day, all doxycycline, non-doxycycline film (*P* < 0.0001), doxycycline (*P *< 0.0001), and non-doxycycline nanofiber dressings (*P *= 0.0001) significantly reduced the IL-1β concentration as compared to the C14 group.

*TNF-α*

(Figure 7B) showed no significant difference between the studied groups in terms of fluctuating TNF-α concentrations on the 3rd and 7th days. On the 14th day, all diabetic rats had a higher TNF-α concentration than the C14 group. Compared to the CD14 group, only the doxycycline nanofiber dressing group (*P *= 0.0003) and non-doxycycline nanofiber dressing group (*P *= 0.0036) significantly decreased the TNF-α concentration. Overall, nanofiber groups showed an improvement in reducing the TNF-α concentration in comparison to the film groups on the 14th day (*P* < 0.05).

*IL-10*

According to (Figure 7C), there were no significant differences between dressing groups with CD groups on days 3, 7, and 14. 

*TIMP-1*

**Figure 8:**
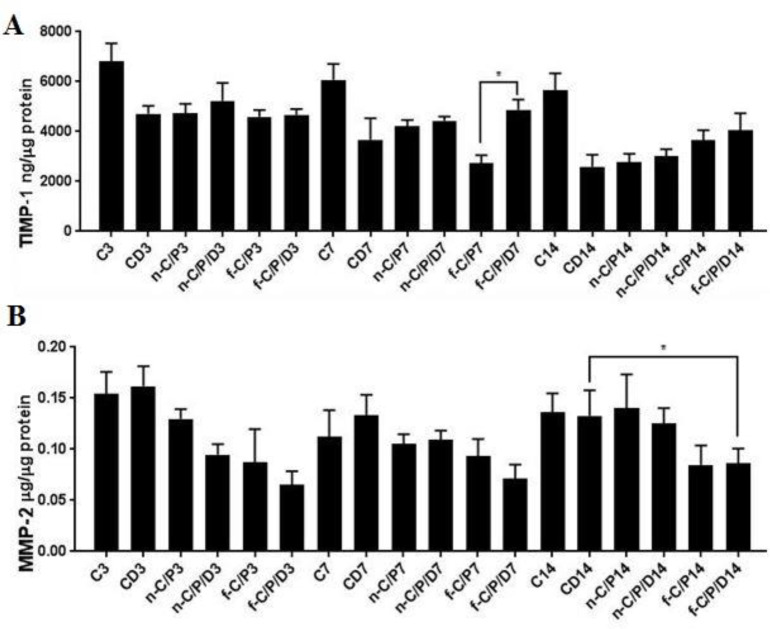
The bar chart Analysis (ELISA). A: TIMP-1 and B: MMP-2 from the studied groups on days 3, 7 and 14. C: Control, CD: Control of diabetes, n-C/P: nanofiber-Chitosan/Polyvinyl alcohol, n-C/P/D: nanofiber-Chitosan/Polyvinyl alcohol/Doxycycline, f-C/P: film-Chitosan/Polyvinyl alcohol, and f-C/P/D: film-Chitosan/Polyvinyl alcohol/Doxycycline. Significance only showed between dressing and CD groups (*: *P* < 0.05)

According to the (Figure 8A), there was no significant difference between the dressing groups in terms of fluctuating TIMP-1 concentrations with CD groups on days 3, 7, and 14. The CD7 group showed a significantly low TIMP-1 concentration than the C7 group on the 7th day (*P* = 0.0248). Also, drug-containing dressings increased the TIMP-1 level as compared to the drug-free state, which was significant only for the film dressing (*P* = 0.0413). 

*MMP-2*

According to the (Figure 8B), the CD14 group showed higher MMP-2 levels than doxycycline film dressing group (*P* < 0.05) on the 14th day. 

## Discussion

In order to evaluate the healing of wound surface area in the rat skin, macroscopic imaging and software-based analysis were used. On the 3rd day, there was no significant difference between the six groups in terms of the percentage of reduction in the wound surface area. The obtained results could be due to the presence of an inflammatory phase of the wound and lack of sufficient time to form the ECM and other compounds involved in the wound healing process. In fact, from the 3rd day, the wound enters the proliferation phase, the most important feature of which is the formation of granulation tissue composed of inflammatory cells, fibroblasts, collagen, fibrin, and fibronectin (34, 35). Also, epithelial tissue, blood vessels, and wound contraction begin at this stage. The results indicate that diabetes can be an indispensable factor in delayed wound healing. The results also indicate that doxycycline along with chitosan can be a useful compound for the healing of chronic diabetic wounds. Alssara reported that the topical gel of chitosan increased the epithelialization, wound size reduction, and granulation tissue formation in rat burn wounds (16).

Investigation of epidermal volume showed that doxycycline dressings increased epidermal volume compared to the diabetic control groups on days 3, 7, and 14.

Our findings showed that all dressings have increased the number of epidermis basal cells as compared to the control and CD groups on days 3, 7, and 14.

Doxycycline has also been able to increase the dermal volume on days 3, 7, and 14.

On the 3rd day, the dressing groups caused a significant increase in collagen volume than the control group. Overall, changes in the collagen volume could not be interpreted properly on the 3rd day due to the delayed entrance of fibroblasts into the wound site. On the 7th day, the only doxycycline film and nanofiber dressing groups were able to significantly increase the collagen volume compared to the C7 and CD7 groups. On the 14th day, doxycycline and non-doxycycline film and also nanofiber dressings showed a significant increase in the collagen volume compared to the C14 and CD14 groups. The results revealed that doxycycline and polymer scaffold acted as a stimulant for increasing skin collagen volume. Adhirajan *et al*. used the doxycycline/collagen combination to treat the wound model in rats. They reported that the amount of tissue collagen and healing rate was significantly higher in the treated groups; moreover, the healing was faster than the control group (36, 37). 

According to the recent researches, it has been shown that neutrophil cells penetrate later into the wound base and remain longer and cause the inflammatory phase prolonged under diabetic conditions (38, 39). In this inflammatory condition, neutrophils and macrophages exhibit lower migration and infiltration to the wound site, and they would remain in place for a longer period and cause chronic wounds (40). Consistent with these studies, our results showed that the neutrophil count in the control group was higher than the CD group on the 3rd and 7th days; however, the same amount was higher in the CD14 group than the C14 group on the 14th day (non-significant). Our results also showed that doxycycline-containing dressings have been able to reduce neutrophil count in all the studied groups.

Our results showed that the lowest number of fibroblasts was observed in the third, seventh, and fourteenth days in the CD group compared to the remaining groups. Furthermore, the number of fibroblasts was higher in all dressing groups than the control group in the entire experiment. Additionally, the doxycycline film and nanofiber dressings groups as compared to the non-doxycycline ones have shown more fibroblasts during the histological evaluation in all days. In one study, in the culture medium, it was shown that the cell migration rate was reduced by 75% in diabetic fibroblasts compared to the normal fibroblasts (41). 

Different vessel length values in diabetes depend on the organ or tissue. Unlike nephropathy and retinopathy, diabetes leads to lower angiogenesis during the wound healing process, which is a factor in delaying wound healing (42). Our results revealed that doxycycline and chitosan have been able to increase the vessel length in the wound tissue and can act as a stimulant for the formation of new vessels in the diabetic wound tissue. 

There are many studies on the tissue, cell, and molecular structure of chronic ulcers, especially diabetic ulcers. The most important goals in these studies were the focus on the expression and protein concentration of the pro-inflammatory cytokines, as well as the MMPs. Mirza *et al*., in a study showed that macrophages isolated from chronic diabetic ulcers elicited the expression of high levels of pro-inflammatory factors in MMP-9, TNF-α, and IL-1β; on the other hand, CD206, IGF-1, TGF-β, IL-10 levels were lower in the wound healing process (43). In addition, it has been observed that the antibody-based inhibition of IL-1β signaling pathway can lead to inhibition of pro-inflammatory agents (MMP-9, TNF-α, IL-1β, and IL-6) and increase the expression of wound healing agents (CD206, IGF-1, TGF-β, and IL-10) (43). Also, the wounds treated by IL-1β neutralizing antibodies led to the better formation of granulated tissue and epithelium in H&E staining; in addition, it showed more collagen deposition in trichrome staining. The researchers suggested that the IL-1β signaling pathway could be a useful therapeutic target for chronic diabetic wounds (43). In a study conducted regarding the role of doxycycline with anti-inflammatory effects, Han *et al*. indicated that doxycycline reduced IL-1β levels (44). In addition to antimicrobial effects, the family of tetracycline antibiotics, in particular, doxycycline, has non-antibiotic effects, including anti-inflammatory effects (45). Many studies have investigated the property of doxycycline at various levels. Based on a study, it was revealed that doxycycline antibiotic could reduce serum IL-1β levels and IL-6; in addition, it could increase the serum level of IL-1 receptor antagonist (IL-1RA) (46). Solomon *et al*. showed that doxycycline reduced the mRNA and IL-1β cytokine protein in the corneal cells (26). Our results showed that doxycycline, non-doxycycline film, and nanofiber dressings have been able to decrease the IL-1β concentration on days 3, 7, and 14, as compared to the C3, C7, C14 and CD3, CD7, CD14 groups non-significantly. Doxycycline dressings elicited the more reduction of IL-1β concentration than non-doxycycline dressings.

In the wound healing process, the TNF-α levels return to its maximum concentration 12 to 24 h after the wound development and then to its normal concentration at the end of the proliferation phase (47). In the early wound healing stages, TNF-α is mainly secreted by the multi-nucleus cells and then by macrophage cells. Many studies have shown abnormal TNF-α concentrations in chronic and diabetic ulcers (47). Elevated TNF-α concentration interferes with the normal wound healing process by inhibiting angiogenesis, cell proliferation, inducing apoptosis or cell death, and inhibiting the migration of cells involved in wound healing by inhibiting SMAD 7 as well as inhibiting the activation of SMAD 2-3 (47). In a study published in 2007, Goren *et al*. reported that the TNF-α inhibitor antibody can improve wound healing and reduce inflammation in rat wounds (48). They suggested that the TNF-α signaling pathway could be an appropriate target for the treatment of chronic wounds, especially diabetic wounds (48). Another research also revealed that doxycycline reduced expression levels of TNF-α protein in the keratinocyte cell culture medium (49). Sun *et al*. reported that doxycycline caused a significant inhibition in TNF-α production and down-regulation of the NF-κB gene (50). Our results showed no significant correlations between TNF-α concentration on the 3rd and 7th days. However, on the 14th day, nanofiber dressing groups had significantly lower levels of TNF-α than the control diabetes group, with doxycycline dressings, in comparison with non-doxycycline dressings, leading to a greater reduction in TNF-α concentration.

One of the cytokines known to have anti-inflammatory activity is interleukin-10 (IL-10) (9). IL-10 has been reported to inhibit the binding of monocytes to endothelial cells and also led to a dose-dependent decrease in the MMP-9 activity. IL-10 also increases TIMP-1 concentration (51). Another study showed that IL-10 stimulated TIMP-1 secretion and inhibition of MMP-2, 9 by prostate tumor cells. It also affects pro-inflammatory cytokines by inhibiting IL-1β expression. Another study showed that pro-and active forms of MMP-2.9 have increased in IL-10^/^rats (52). Results of a study on the human monocyte cell culture showed that doxycycline increases the level of IL-10 from human monocyte cell culture (53). Our results indicated that doxycycline could increase the IL-10 level compared to the CD3, CD7, and CD14 group (non-significant).

A review of 94 patients with diabetic foot ulcers with the aim of measuring serum MMP-2, MMP-9, TIMP-1 and TIMP-2 levels in a 12-week treatment period showed that the mean serum concentrations of MMP were lower in the first visit of patients with proper healing than those with poor tissue healing (124.2 μg/L vs. 374.6 μg/L, *p* < 0.05) and the amount was 5 times lower at the end of the fourth week. They suggested that the MMP/TIMP ratio could be an appropriate predictive factor for the healing of chronic diabetic foot ulcers patients; therefore, it could be a new target for the treatment of these ulcers in the future (54). Liu *et al*. investigated the MMP and TIMP concentration, as well as the MMP/TIMP ratio in human diabetic wounds. They showed that MMP concentration and MMP/TIMP ratio is higher in unhealed wounds than the healed diabetic wounds. They suggested that this ratio could be used as a predictor to heal diabetic wounds (55). Doxycycline has been shown to significantly reduce endometrial MMP and increase TIMP-1 levels; furthermore, it has also been shown that this antibiotic has significantly reduced MMP-9 concentration in serum and tissue (56, 57). Using doxycycline dosage as SDD (sub antimicrobial dose doxycycline) for the treatment of periodontitis, Chouy *et* *al*., found that doxycycline reduced the MMP-9 concentration and increased TIMP-1 concentration (58). The results of the present study indicated that doxycycline increased the TIMP-1 concentration in comparison to the other groups. The results of the present study on changes in MMP-2 concentrations showed that doxycycline reduced MMP-2 levels. Many experiments have shown that the differentiation and proliferation of fibroblasts are impaired; in addition, collagen and dermal hyaluronic acid production is reduced under diabetic conditions (59). Also, the concentration of pro-inflammatory cytokines and growth factors (IL-1, IL-6, TNF-α, PDGF and CTGF), and MMPs (MMP-2, 3, 9, 13) increased by fibroblasts under these conditions (59). According to the recent studies, they have also shown that NF-κB levels have increased under diabetic conditions and NF-κB is an essential factor in inhibiting the proliferation, differentiation, and migration of tissue keratinocytes (59). In one study, MMP-2,8,9 was less active in the doxycycline treatment group than the control group (36). Sarah *et al*., evaluated the effect of oral doxycycline dose (20 mg/day) in patients with chronic venous ulcers for three months. They found that the doxycycline-treated group showed faster healing and also inhibited MMP-9 (60). 

## Conclusion

It was ultimately attained that doxycycline dressings performed better than non-doxycycline dressings; in addition, the doxycycline dressings had a better outcome in evaluated epidermis size, collagen volume, neutrophil count, fibroblast count, basal epidermal cells, vessel length, IL-1β, TNF-α, TIMP-1, and MMP-2 concentrations. It seems that doxycycline improved wound healing by decreasing the proinflammatory cytokines and matrix metalloproteinase as well as TIMP increase. We suggest the use of nanofiber wound dressing in the early phase of wound healing and film wound dressing in the proliferation phase.
